# Matrix Intensification Alters Avian Functional Group Composition in Adjacent Rainforest Fragments

**DOI:** 10.1371/journal.pone.0074852

**Published:** 2013-09-13

**Authors:** Justus P. Deikumah, Clive A. McAlpine, Martine Maron

**Affiliations:** Landscape Ecology and Conservation Group, School of Geography, Planning and Environmental Management, The University of Queensland, Brisbane, Queensland, Australia; James Cook University, Australia

## Abstract

Conversion of farmland land-use matrices to surface mining is an increasing threat to the habitat quality of forest remnants and their constituent biota, with consequences for ecosystem functionality. We evaluated the effects of matrix type on bird community composition and the abundance and evenness within avian functional groups in south-west Ghana. We hypothesized that surface mining near remnants may result in a shift in functional composition of avifaunal communities, potentially disrupting ecological processes within tropical forest ecosystems. Matrix intensification and proximity to the remnant edge strongly influenced the abundance of members of several functional guilds. Obligate frugivores, strict terrestrial insectivores, lower and upper strata birds, and insect gleaners were most negatively affected by adjacent mining matrices, suggesting certain ecosystem processes such as seed dispersal may be disrupted by landscape change in this region. Evenness of these functional guilds was also lower in remnants adjacent to surface mining, regardless of the distance from remnant edge, with the exception of strict terrestrial insectivores. These shifts suggest matrix intensification can influence avian functional group composition and related ecosystem-level processes in adjacent forest remnants. The management of matrix habitat quality near and within mine concessions is important for improving efforts to preserveavian biodiversity in landscapes undergoing intensification such as through increased surface mining.

## Introduction

The conversion of tropical forests by human activities to other land use systems is one of the greatest impacts on biodiversity [1]. This process has introduced human-dominated matrices surrounding native forest remnants [2] and increased the isolation distance among these remnants [3]. The influence of such matrices can be pervasive throughout the landscape, such that changes occurring in the matrix may not only reduce matrix habitat suitability, but also may introduce movement barriers and alter the biophysical conditions within adjacent remnants [4,5]. These changes may lead to loss of biodiversity and, consequently, shifts in ecosystem functionality [6,7].

The link between biodiversity loss and ecosystem functioning depends on the range of functional roles of species, rather than species identity [8], because different species can perform similar ecological roles [9]. Thus, changes in species numbers (decrease or increase) do not necessarily imply changes in functional diversity [10]. Conclusions about ecosystem functionality from studies of change in species richness alone may therefore be of limited validity [11]. As such, effective conservation of degraded ecosystems requires understanding of the effects of landscape change on species richness and also on the functional diversity and abundance within remnant habitats [12,13].

Conversion of the typically low-intensity agricultural lands found in many parts of the tropics to high-intensity surface mining represents a significant increase in patch-matrix contrast. Such changes may cause shifts in faunal communities not only at the affected sites but also within nearby remnants. Intensification in the surrounding matrix can affect species occupancy of remnants by affecting inter-patch movement and patch colonization [14], foraging habitat within patches [15], population sizes [16,17], and interspecific interactions within patches [18,19]. These changes can lead to more homogenous communities [20], favouring the dominance of generalists while decreasing occurrence of rare species and specialists [21]. Thus, with persistent matrix changes surrounding native remnants, some species may benefit (“winners”) and expand their geographic range to replace rare and disturbance sensitive species (“losers”) [22].

Mining is an important contributor to the economy of many countries, contributing > 45% of global GDP [23] and responsible for > 67% of the GDP of developing countries [24-26]. Ever-larger land areas have been claimed for the purposes of mining leading to the loss of both native vegetation and arable lands [26]. Although the conversion of farmlands to other land uses is less damaging to biodiversity than conversion of forests, such changes can still influence local biodiversity. Native remnants formerly surrounded by low intensive land-use matrices increasingly are embedded in less-hospitable, higher-intensity matrices dominated by surface mining [27]. Consequently, biodiversity may be less likely to persist in such landscapes even if remnant native vegetation remains intact. This may lead to a shift in ecosystem functioning, if functional groups are differentially affected by landscape change [13].

Despite the potential impact of matrix intensification on the conservation of biodiversity and ecosystems [28], only a few studies have reported the importance of forest modification on changes in diversity and abundance within vertebrate functional groups [29-33]. It remains unknown how functional groups and community structure of fauna in native remnants are affected by increasingly common matrix intensification in the tropics. Such understanding of faunal responses to the growing replacement of lower-intensity with higher-intensity matrix land uses is necessary to develop predictions of shifts in ecosystem function, and to determine how best to mitigate undesirable impacts.

Here we evaluated the effect of matrix type and local-scale habitat factors on avian community composition and abundance within functional guilds. Birds are the best known vertebrate group of organisms [34] and they provide important ecosystem services such as seed and fruit dispersal, pollination, nutrient deposition and pest control and are critical agents in tropical forest regeneration [30]. We compared avian assemblages in fragments adjacent to two contrasting matrix types (mining vs. agricultural) at two distances from remnant edge (edge/interior) in south-west Ghana. We hypothesized that intensification of adjacent matrices results in a shift in functional composition of avifaunal communities, and will have a homogenising effect within functional groups.

## Methods

### Ethics Statement

All field work was approved by the University of Queensland Animal Ethics Committee under permit number GPEM/191/10. Permission to access conservation reserves was granted by the Forestry Commission and the Wildlife Division of Ghana. Permission to access private company properties (e.g. mining sites adjacent reserves) was granted by relevant authorities.

### Study area

The study was conducted in the fragmented upper Guinea forest, west Ghana. Located along the Gulf of Guinea in west Africa (3°5`W-1^°^ 10`E; 4°35`N-11^°^N), Ghana has a total landmass of 238,500 km^2^. Ghana extends over four main biogeographic zones: the Guinea-Congolian in the south-west, the Sudan in the north, the Guinea-Congolian/Sudanian transition zone in the centre and the south-east, and the Volta in the east [35]. The forest areas are confined to the Guinea-Congolan zone and covers an area of approximately 9.2 million hectares. Most of the tropical tall forests are in the southwestern part of the country but are highly fragmented as a result of clear-fell logging for high-value timber products and rapid human population growth. These areas are also ideal climates for raising cash crops and food crops, and are exposed to recurring annual fires [35]. The forest fragments of south-west Ghana are surrounded by a land use matrix dominated by small farms and fallow land with relictual native forest trees retained within these croplands [35,36].

The area is also rich in minerals such as gold, bauxite, and iron ore, and their extraction is a serious threat to the region’s forests [35]. Many large-scale surface gold mining operations have recently been established, often located adjacent to forest reserves [23,37,38]. Ghana, Africa’s second largest producer of gold, has attracted many international investors and economic success over the past three decades [39]. This has led to a gold rush among local inhabitants who have become highly dependent on this industry for their livelihood, but with serious environmental consequences [26]. Mining in Ghana, has degraded the environment and transformed many natural habitats [37,38] with land acquisition for mining targeted at both natural forests and farmlands. In the Wassa district of south-western Ghana alone, a total of 8103 ha are under surface mining; of this, 4935 ha is converted farmlands with the remainder former human settlements and forest [26]. Consequently, patch-matrix contrast has increased with the replacement of the relatively lower-contrast farmland matrix which was more structurally heterogeneous and thus more similar to vegetation in nearby patches [40].

### Experimental design and bird surveys

**Figure 1:**
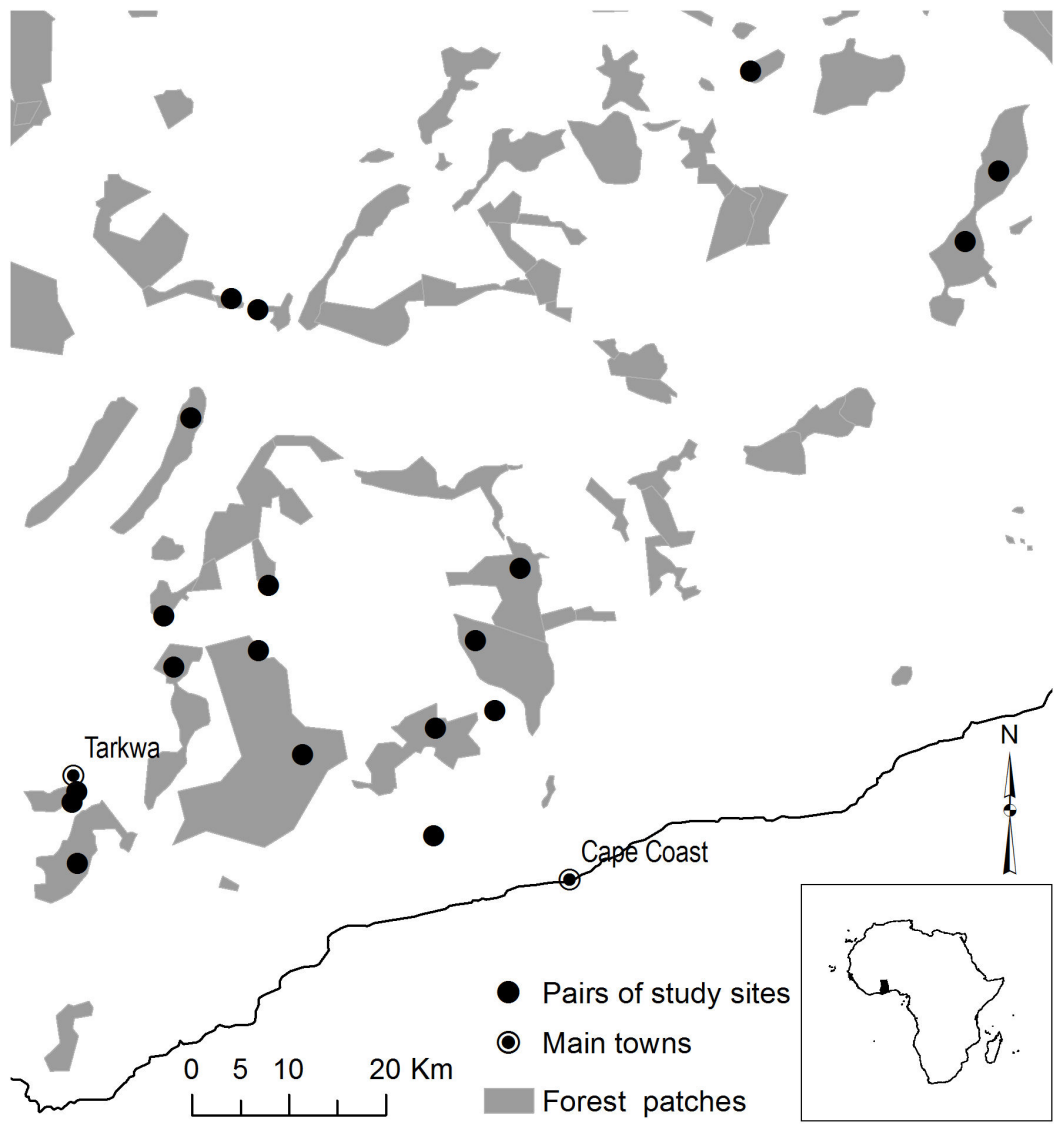
Map of South-west Ghana showing bird survey locations within tropical forest remnants. Insert in bottom right shows the location of Ghana in Africa.

Thirty-two sites were selected in 16 patches of forest ranging in size from 200-58,800 ha. In each patch, one site was located at the edge (within 50 m of the forest boundary, classified as “edge” sites) and one closer to the interior (at least 500 m from the forest boundary, classified as “interior” sites) (see Figure 1). Sixteen of these sites were located adjacent to a matrix dominated by active surface mining areas, and 16 were adjacent to a matrix dominated by croplands. The dominant matrix type was defined as the land use type in > 85% of the matrix within a 1 km buffer of the study patch.

At each site, three sample stations were located 200 m apart. Birds were surveyed at each station two times in the dry season (November–March) and two times in the wet season (June–September) from 2010 to 2011. The point count method [41,42] was used to record all bird contacts (sightings and vocal calls) within a 50 m radius [41]. All bird surveys were conducted by the same person (JPD) to avoid observer bias in both identification and distance estimation. The total number of individuals of each species detected at each sample station was also recorded and used to create an abundance database. Each point count lasted for 20 minutes and counts were conducted on two occasions during the morning (0530 −1100 h) and once in the afternoon (1430-1700h), coinciding with the main feeding times of birds [42]. Before each count, the observer allowed 5 minutes for birds to settle following the initial disturbance caused by the observer. Efforts were made to avoid double counting of individuals moving among stations. Counts were not undertaken on days with bad weather conditions (windy, misty, or rainy).

### Classification of avian functional groups

The ecological attributes of recorded bird species were identified based on existing literature. The system by Bennun et al. [43] to classify birds of Kenya and Uganda was adopted with each species assigned to one of four categories based on their habitat preference: forest-dependent (specialists), forest generalist, forest visitors, and open habitat species. All species also were grouped according to six exclusive food preference categories (carnivores, frugivores, granivores, insectivores, nectarivores, and omnivores (based on information in ‘The Birds of Africa’ Vols. 1-7) [33,44-46]. Birds in each food preference category were further divided where possible; for example, frugivores were further categorised as obligates (depending entirely on fruits) and partial/opportunistic frugivores (feeding on different food items including fruits and insects). Thus, the functional guilds used in these analyses were identified based on combining information on forest habitat and food preferences, foraging strategy and main foraging stratum of each bird [47].

### Landscape and vegetation surveys

Forest patches were manually digitised from 1:50,000 Google, Earth image (October 2012). We also classified and mapped cropland, abandoned farmlands and surface mining areas. The total area of each forest patch was calculated by manually digitizing from 1:50,000 Google, Earth Maps (October 2012) using ArcGIS 10 [48]. We calculated the area of each forest patch containing a bird survey site and measured linear distance from each forest patch surveyed to the nearest largest patch. We also calculated the total area of forest habitat within 1 km buffer distance from each bird survey site (Table 1).

**Table 1:** Description of explanatory variables in addition to adjacent matrix type and distance to edge used to assess influence of site and landscape characteristics on forest bird richness.

**Variable**	**Units**	**Description**
Forest extent*	km2	Amount of forest habitat in 1km^2^ radius of survey site.
Forest type*	−	Type of tropical rainforest based on the total annual rainfall received (moist semi-deciduous or evergreen)
**Shrub density**	percent	Understorey foliage projected cover of small plants and young trees (with DBH < 10 m)
Fruiting trees*	count	Sum of all fruiting vegetation (trees, shrubs and lianas) across all surveys.
**Flowering trees**	count	Sum of all flowering vegetation (trees, shrubs, lianas) across all surveys

* Explanatory variables used in analyses in addition to matrix type and distance to edge

Vegetation surveys were conducted to characterise the structure and composition of the vegetation at each site. Trees with diameter at breast height (dbh) > 60 cm (large trees) were counted in five randomly placed 20 x 20 m quadrats at each site. Within the same 20 x 20 m quadrats, we counted all fruiting and flowering plants. Within five randomly selected 5 x 5 m quadrats, we visually estimated ground cover, including grass, litter and bare ground at each site. Where appropriate, all measurements were standardised to values per hectare or per square metre [49]. The logging history of each forest patch was assessed from literature [35,50].

### Statistical analyses

#### *Functional diversity and abundance*

In a first analysis, we compared the mean abundance per survey of birds in all 19 functional guilds recorded between sites near surface mining and those near agriculture areas, and between the two distances from remnant edge. After initial exploration of the abundance data for normality, correlation and equality of variance, Type II ANOVA was considered suitable and was used to compare the abundance of bird functional groups among treatments. Second, we computed functional evenness, *Evar* (measure of species evenness) at each site for all functional groups using the formula:

$E\mathrm{var}=1-2/\pi \arctan\left\{{\sum_{t=1}^{s}In(x_{i})-\sum_{t=1}^{s}\left(\frac{In(x_{i})}{s}\right)}^{2}\frac{1}{s}\right\}$,

where *x*_*i*_ = abundance value for species *i* and *S* = species richness. Evenness values for all functional groups were compared using Type II ANOVA.

#### *Multivariate analyses*

Multivariate data analyses were employed to examine variation in avian assemblage composition between and within edge proximity and matrix categories. Non-metric multidimensional scaling (NMDS) ordination was conducted using Bray-Curtis dissimilarity matrices in PRIMER 6 software to represent visually the bird assemblages among matrix and context groupings [51]. This was followed by a vector fitting protocol performed in R vegan package [52] to examine which standardized environmental variables were associated with variation in the bird assemblages in the NMDS ordination. Vector fitting can reveal the most important environmental variables contributing to the observed pattern of bird assemblages in the study area [49]. Prior to vector fitting, all explanatory variables were tested for collinearity using Spearman’s correlation coefficient. Pairs of explanatory variable with high correlation can be considered as proxies of one another (Booth et al. 1994). The explanatory variable in a correlated pair (coefficients of correlation, r > | 0.5|) that most plausibly would influence bird assemblages and community composition was retained for the final analyses (Appendix Table S5).

To test for differences in community composition among distance from edge and matrix groups, we used two-way crossed ANOSIM with replicates to compare within-group similarities and between-group dissimilarities with 100 permutations using PRIMER 6.0 [51]. In this analysis, sites were considered as samples and average abundance of each species as dependent variables while distance from edge (edge/interior) and matrix type (mining/agricultural) were factors.

SIMPER (similarity percentages) analysis was carried out in PRIMER 6 (Plymouth Marine Laboratory) to determine the role of individual species in contributing to the differences between groups. This allowed us to determine individual species most responsible for the average percentage similarities within and dissimilarities between context and matrix groups [53].

Finally, we conducted a compositional indicator species analysis (ISA) using the labdsv package in R [54] to identify bird species indicative of each group. Indicator species analysis permits statistically rigorous assessments of which species characterize a given ecosystem [55].

## Results

### Functional diversity and abundance

A total of 7, 257 individuals of 195 species from 46 families were detected including 34 migrants (comprising 18 intra-African, 9 seasonal and 8 Palearctic migrants). Mean total abundance of birds differed significantly between matrix types but not with distance from the remnant edge. Mean abundance of members of avian functional guilds also differed significantly between matrix types, with mean values generally higher in sites adjacent to agriculture than those in surface mining matrices (Table 2). The direction of the differences in abundance between edge and interior site, however, was functional group-specific. Matrix type alone had a significant negative effect on abundance of four guilds: obligate frugivores, all lower and upper strata birds, and lower strata foliage gleaners. Distance from edge had a significant influence on lower strata bark gleaners, upper strata foliage gleaners and salliers, with higher abundances of each group recorded at interior than at edge sites (Table 2). Both matrix type and distance from edge significantly influenced the abundance of strict terrestrial insectivores, all bark gleaners and upper strata bark gleaners (Table 2).

**Table 2:** Results of ANOVA comparisons of abundance within avian functional guilds at different distances from edge and matrix types.

**Functional group**	**Agricultural matrix (mean ± SD**)		**Mining matrix (mean ± SD**)		***F*- statistics**		
	Edge	Interior	Edge	Interior	Dist.edge	Matrix	Interaction
**Strict terrestrial insectivores**	71.0 (15.6)	90.5 (13.4)	54.3 (25.6)	62.8 (20.9)	4.13*	10.44*	6.64*
**Canopy insectivores**	5.6 (3.3)	8.1 (2.4)	7.6 (2.9)	6.6 (3.4)	0.49	0.05	2.67
**All lower strata birds**	130.6 (57.7)	119.8 (47.9)	97.9 (34.4)	61.0 (19.1)	2.49	9.45**	0.73
**All upper strata birds**	125.4 (34.2)	140.6 (21.7)	115.5 (17.4)	107.5 (34.9)	0.13	4.68*	1.37
**Lower strata bark gleaners**	2.3 (1.4)	4.3 (1.3)	1.3 (2.9)	2.8 (1.7)	6.59*	3.36	0.13
**Salliers**	2.00 (1.9)	5.2 (4.3)	1.5 (1.2)	2.4 (1.4)	5.07*	3.35	1.61
**Upper strata bark gleaners**	3.5 (3.2)	5.5 (3.1)	1.3 (1.3)	2.8 (1.4)	4.21*	8.59**	0.09
**Upper strata foliage gleaners**	10.0 (3.3)	16.4 (7.6)	10.9 (4.4)	12.9 (4.2)	4.71*	0.74	1.81
**Bark gleaning insectivores**	0.8 (1.0)	2.1 (1.7)	0.3 (0.7)	0.8 (0.9)	5.27*	5.27*	1.15
**Obligate frugivores**	22.9 (16.8)	21.3 (14.3)	12.0 (2.4)	15.5 (8.2)	0.05	3.98*	0.39
**Raptors**	5.6 (3.3)	8.1 (2.4)	7.6 (2.9)	6.5 (3.4)	0.49	0.05	2.67
**Granivores**	25.1 (31.5)	12.9 (15.9)	9.9 (11.6)	7.0 (9.2)	1.25	2.44*	0.48
**Omnivores**	32.4 (16.1)	31.1 (8.3)	31.4 (14.1)	29.3 (15.9)	0.12	0.08	0.01
**Nectarivores**	1.0 (0.8)	2.0 (1.4)	1.5 (1.3)	1.4 (1.9)	1.08	0.02	1.78
**Partial frugivores**	137.6 (100.5)	127.6 (86.0)	72.0 (14.1)	93.3 (49.5)	0.05	3.88	0.39
**Lower strata aerial sweepers**	8.4 (3.9)	7.5 (2.3)	6.6 (2.1)	7.1 (4.1)	0.03	0.87	0.36
**Lower strata foliage gleaners**	8.9 (3.1)	10.3 (3.4)	7.0 (3.2)	5.3 (3.2)	0.03	9.07**	3.55*
**Upper strata aerial sweepers**	13.5 (6.1)	11.0 (5.5)	11.3 (3.6)	11.1 (7.9)	0.39	0.25	0.32

Significance codes: <0.001 '*** '; < 0.01 '** ' <0.05 '* '

Functional evenness of seven functional groups differed significantly with matrix type and proximity to edge (Table 3). Evenness was higher for four of these guilds (obligate frugivores, strict terrestrial insectivores,all lower and upper strata birds and all bark gleaners) in sites near agricultural matrices than mining matrices, except for granivores which were more even in sites adjacent to mining matrices. Evenness of strict terrestrial insectivores was lower in remnants adjacent to mining matrices but higher at interior sites compared to edges for both matrices. Proximity to patch edge alone significantly affected evenness of raptors and lower strata aerial sweepers with both groups more evenly distributed at interior sites than edges (Table 3).

**Table 3:** Results of ANOVA (Type II tests) in which species evenness, H, of avian functional groups were modelled with distance from edge, matrix and interaction between both.

Functional group	Agricultural matrix(mean ± SD)		Mining matrix (mean ± SD)		*F*- statistics		
	Edge	Interior	Edge	Interior	Dist.edge	Matrix	Interaction
Terrestrial insectivores	0.74 (0.08)	0.79 (0.04)	0.48 (0.30)	0.69 (0.10)	5.23*	9.87**	2.11*
All lower strata birds	0.77 (0.12)	0.71 (0.08)	0.59 (0.14)	0.55 (0.15)	1.42	14.16***	0.05
All upper strata birds	0.53 (0.19)	0.43 (0.10)	0.36 (0.12)	0.37 (0.15)	0.68	4.78*	0.96
Obligate frugivores	0.67 (0.16)	0.61 (0.12)	0.44 (0.31)	0.34 (0.29)	0.90	8.37**	0.10
Partial fruigivores	0.72 (0.12)	0.67 (0.06)	0.66 (0.13)	0.72 (0.07)	0.07	0.00	2.03
Salliers	0.19 (0.12)	0.18 (0.12)	0.22 (0.19)	0.18 (0.18)	0.17	0.08	0.05
Upper strata bark gleaners	0.59 (0.40)	0.48 (0.39)	0.75 (0.37)	0.60 (0.31)	1.04	1.23	0.03
Upper strata foliage gleaners	0.26 (0.18)	0.27 (0.05)	0.17 (0.12)	0.27 (0.11)	1.84	1.16	0.93
All bark gleaning insectivores	0.59 (0.40)	0.40 (0.40)	0.83 (0.36)	0.60 (0.31)	2.06	2.31	0.13
Lower strata bark gleaners	0.89 (0.02)	0.99 (0.01)	0.70 (0.43)	0.64 (0.42)	0.08	9.78**	0.08
Lower strata foliage gleaners	0.30 (0.12)	0.38 (0.06)	0.27 (0.10)	0.34 (0.14)	3.59	0.84	0.01
Canopy insectivores	0.41 (0.40)	0.10 (0.07)	0.27 (0.16)	0.30 (0.32)	2.06	0.07	3.05
Raptors	0.41 (0.30)	0.74 (0.38)	0.50 (0.41)	0.81 (0.35)	6.15*	0.38	< 0.01
Granivores	0.61 (0.45)	0.67 (0.39)	0.13 (0.20)	0.13 (0.07)	0.08	21.18***	0.06
Omnivores	0.20 (0.11)	0.24 (0.11)	0.18 (0.12)	0.20 (0.10)	0.49	0.42	0.04
Nectarivores	0.79 (0.32)	0.75 (0.36)	0.92 (0.22)	0.90 (0.19)	0.11	2.00	< 0.01
All insectivores	0.42 (0.11)	0.46 (0.06)	0.42 (0.04)	0.39 (0.13)	0.04	1.21	1.11
Lower strata aerial sweepers	0.19 (0.12)	0.42 (0.27)	0.10 (0.15)	0.35 (0.32)	5.29*	0.19	0.24
Upper strata aerial sweepers	0.19 (0.18)	0.14 (0.09)	0.13 (0.06)	0.35 (0.41)	0.94	0.90	2.69
All aerial sweepers	0.34 (0.08)	0.21 (0.21)	0.29 (0.10)	0.31 (0.13)	1.26	0.33	2.39

Significance codes: <0.001 '*** '; < 0.01 '** ' <0.05 '* '

### Community composition

**Figure 2:**
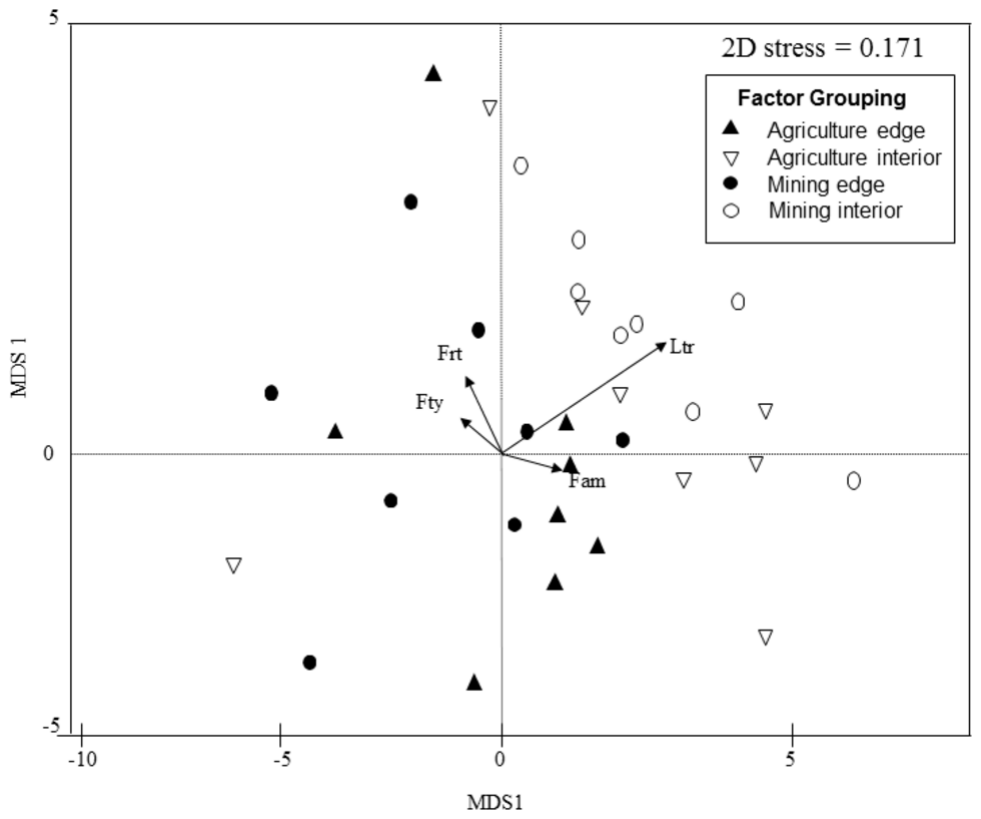
Non-metric multidimensional scaling ordination of proximity to edge and matrix type by key habitat attributes according to their dissimilarities in species composition within a two-dimensional association space. Fitted arrows indicate environmental variables with significant importance in structuring avian communities (***Ltr*** = density of large trees, ***Frt*** = density of fruiting trees, ***Fam***(1 km^2^) = amount of forest habitat in 1km^2^ radius and ***Fty*** = forest type). *Arrow* direction indicates direction of steepest increase in respective variable, *arrow* length indicates precision of inference and angles between *arrows* and axes reflect their correlations.

The results of the NMDS ordination of species composition showed strong clustering of sites according to proximity to edge and weak clustering to matrix types (Figure 2). Significant differences were observed in community composition between edge proximity (Global R = 0.402, p = 0.001) and matrix type (Global R = 0.205, p = 0.001) groups in a two-way crossed ANOSIM with replicates. The R-statistic for the matrix group comparison was relatively low (< 0.25) indicating separation in community structure between groups is relatively weak [56].

Vector fitting of environmental variables to the bird assemblage NMDS ordinations from permutation analysis showed significant correlation of three environmental covariates: amount of forest habitat in 1 km radius (p = 0.043), number of fruiting trees (p = 0.041) and density of large trees (p = 0.013) with avian species composition. Goodness of fit p-values are based on 99 permutations (Figure 2).

Given the ecological importance of both distance from edge and matrix type in structuring avian communities, SIMPER analysis was carried out to assess the species groups that defined dissimilarity between distances from edge (edge/interior) and matrix groups (mining/agricultural). The results of functional groups contributing 90% of the dissimilarity between treatment groups are presented in appendix Table S1. Average abundance of partial or opportunistic frugivores was substantially higher in edge sites compared to interior sites, contributing 26% of the between-group dissimilarity. Average abundance of frugivore-insectivores was also substantially higher in remnants near agricultural matrix than those near mining matrix and this difference contributed 26% of the dissimilarity between matrix groups. Strict terrestrial insectivores contributed >41% each to average dissimilarities while forest specialists contributed over 37% and 40% respectively to distance to edge and matrix groups dissimilarities (Table S1).

Results of compositional indicator species analysis revealed seven species with significant indicator values for interior sites, all of which are forest specialists and two of which (green-tailed bristlebill, *Bleda* *eximia* and rufous-winged Illadopsis, *Illadopsis* *rufescens* ) are species of conservation concern. Species indicative of edge sites were mainly forest generalists and visitors (Table S2). A total of 11 species recorded in remnants near farmlands had significant indicator values, of which seven were forest specialists. Only four species were significant indicators for remnants located near adjacent surface mining, of which two (Yellow-throated Tinkerbird, *Pogoniulus* *subsulphureus* and Red-fronted Ant-pecker, *Parmoptila* *rubrifrons* ) were forest specialists (Table S3). Complete list of significant bio-indicator species characteristic of both groups is presented in appendix Tables S2 & S3.

### Variation in vegetation characteristics

Three key site-level vegetation variables used in the analysis differed significantly with matrix type and proximity to edge (Table S4). In general, there were more fruiting trees in remnants embedded in agricultural landscapes, particularly near forest edges, suggesting higher productivity at agricultural edges [33]. However, sites located near patch edges in mining landscapes had fewer fruiting trees than those located far from the edge (Table S4).

## Discussion

Matrix intensification and proximity to remnant edge significantly influenced the abundance and evenness of several different functional groups. We found that both factors strongly influenced the representation within the assemblage of species with particular dietary preferences and foraging strategies. Mean abundance and evenness of obligate frugivores, terrestrial insectivores, lower and upper strata birds as well as foliage and bark gleaning insectivores were all significantly lower in remnants adjacent to surface mining sites compared to their remnants in agricultural landscapes. Our study suggests a significant shift in functional composition of avian communities adjacent to highly-intensive mining matrices. This implies that even with no further tropical forest loss in fragmented Upper Guinea forest landscapes, the conversion of low contrast agricultural lands to high intensive surface mining may have significant negative consequences for tropical forest ecosystem functioning.

### Matrix and edge effects on foraging guilds

Overall, the composition of different functional groups was significantly influenced by matrix intensity and proximity to remnant edge. Mean abundance of obligate frugivores, strict terrestrial insectivores, upper and lower strata birds and foliage gleaning insectivores was lower in remnants adjacent to surface mining sites than those in agricultural sites. This may be attributed to a lack of important habitat resources in the mining landscape for these birds. Tropical agro-ecosystems often retain many remnant trees [33,57-59]. Apart from providing food resources to forest birds that move into the matrix itself, such trees act as connecting stepping stones for inter-patch movements [60], and also can provide nesting sites and roosting places [60,61]. Due to the lower patch-matrix contrast, agricultural matrices may reduce the edge effects created by landscape modification [62], and improve landscape connectivity [63]. In our study, sites adjacent to mining landscapes had fewer fruiting trees, which may have resulted in lower abundance of obligate frugivores [64]. The lower abundance of insectivores in mining landscapes, may be related to chemical pollution from mining activities (arsenic, mercury, DDT and other organochlorides) that can reduce insect biomass near and within remnants [65].

We found that mean evenness was lower in remnants adjacent to mining for obligate frugivores, strict terrestrial insectivores, all lower and upper-strata birds and bark gleaners. In most cases, the effect was evident regardless of the distance from remnant edge, with the exception of strict terrestrial insectivores. Thus, a small number of species within these groups dominate bird assemblages in mining sites compared to sites adjacent to agriculture (function homogenization) [66]. We found that while remnants adjacent to surface mining matrices may have become unsuitable for rare specialists and frugivores (“losers”), widespread generalists and open country species (“winners”) that may have expanded their geographic range remain [22,66]. This process of homogenization within functional groups can lead to altered ecosystem services and effect functioning of tropical ecosystems within surface mining landscapes [30,66].

Strict terrestrial insectivores, bark gleaners, upper strata foliage gleaners and salliers were more abundant and their evenness was greater in interior sites compared to those at forest edges. The density of large trees and amount of forest habitat within 1 km of sites were greater for sites in the interior of remnants in both matrix types, and density of fruiting trees was higher at edge sites in agricultural landscapes. Clough [32] found that increasing tree cover in cacao plantations in Sulawesi led to increased species richness of frugivores and insectivores. Such sites may provide more conducive microclimates for many invertebrates and insects that may in turn attract insectivorous birds [67]. Differences in abundance of upper-strata foliage and bark-gleaning insectivores may be the result of higher productivity of herbivorous insects in the upper canopy where primary productivity is higher due to higher light intensity received [67].

Reduction in the abundance and evenness of strict terrestrial insectivores at agricultural edges may be as a result of significant reduction of insect food resources at the lowest stratum at edges due chemical pollution. In Ghana, 87% of farmers use some form of agrochemicals to control weeds and pests as well as increase yield [68,69]. Herbicides such as N,N′-dimethyl-4,4’-bipyridinium dichloride (paraquat) are highly toxic to animals with serious and irreversible effects [70] but are heavily used in our study landscapes. Mass spraying of cocoa ( *Theobroma* *cacao* ) by government and individual farmers to increase cocoa production and reduce pests is a common activity in Ghana, particularly in the study area [71].

### Potential consequences for ecosystem function

Changes in functional evenness and abundance can influence ecosystem level processes independently of species richness (Dangles & Malmqvist 2004). Matrix change due to the replacement of low-intensity agricultural lands with higher-contrast surface mining affects bird species groups, potentially via alteration of vegetation structure and composition and chemical pollution in fragmented landscapes of southwest Ghana. The influence of these disturbances varies among species and functional groups, resulting in perturbed assemblages with altered representation within different functional groups. In this study, obligate frugivores, terrestrial insectivores and insect-gleaners were most affected functional groups.

Frugivores and many insectivores perform critical roles in ecosystem function through pollination, fruit and seed dispersal, control of herbivorous insects, and regeneration of tropical forests [6,30,72]. Studies show that many tropical rainforest plant species decline in fragments due to loss of dispersers such as large frugivores [73,74]. Therefore, reduction in frugivorous birds may have significant negative impacts on forest regeneration.

Foliage gleaners assist in the control of herbivorous insect populations [75] and may also provide services such as pollination and dispersal while gleaning for insect food [76]. Therefore, low diversity and abundance among these groups in remnants near mines implies key ecological processes within tropical forest landscapes may be disrupted. Reduction in pollination and dispersal can negatively affect long-term regeneration of remnant forests [77]. Our results suggest that apart from the ecological processes identified in this study, matrix intensification impacts can potentially cascade through other trophic levels such as shifts in predation pressure on invertebrates within fragmented tropical forest landscapes.

Greater diversity within functional groups may lend stability to ecological functioning [78]. For frugivores, different species within this group target different fruiting resources, and so their functions are often complementary [79,80]. High species richness within functional groups implies greater functional redundancy. When functional redundancy is high, if one species is lost or declines, there are more likely to be other species to perform the service it was performing [81]. Therefore, functional homogenization within these groups in surface mining areas can have negative impacts on the functioning of tropical ecosystems.

We conclude that surface mining adjacent to tropical forest remnants results in negative consequences for several functional groups of birds compared to the agricultural matrices it often replaces [82]. Thus, even without further loss of native forest, conversion of these farmlands to surface mining can impact on tropical forest ecosystems and its dependent biodiversity. Conversion to surface mining in fragmented tropical forest landscapes is itself a conservation issue deserving of attention. Management strategies should focus on improvement of matrix habitat quality by identifying priority areas for restoration adjacent to existing remnants. Retaining native patches and scattered large trees on mines concessions may soften the matrix and help preserve avian biodiversity in surface mining landscapes of south-west Ghana.

## Supporting Information

Table S1 **Results of SIMPER analysis showing mean abundance of species groups responsible for dissimilarity and percentage contribution between both dist. edge and matrix groups.**
(DOCX)

Table S2 **Significant bio-indicator species characterizing edge and interior communities.**
(DOCX)

Table S3 **Significant bio-indicator species characteristic of remnants located in two matrix types.**
(DOCX)

Table S4 **Results from two-factor ANOVA comparing explanatory variables between distance to edge and matrix categories.**
(DOCX)

Table S5 **Correlation matrix of explanatory variables.** Coefficients in **bold** shows highly correlated variables that were excluded in the analyses.
(DOCX)
