# Supplementary material for: Matrix Intensification Alters Avian Functional Group Composition in Adjacent Rainforest Fragments
Source: PLoS One. 2013 Sep 13;8(9):e74852. doi: 10.1371/journal.pone.0074852 (PMC3772896; doi:10.1371/journal.pone.0074852)
Supplement: Table S1 — Results of SIMPER analysis showing mean abundance of species groups responsible for dissimilarity and percentage contribution between both dist. edge and matrix groups. (DOCX) [file pone.0074852.s001.docx]

Table S1: Results of SIMPER analysis showing mean abundance of species groups responsible for dissimilarity and percentage contribution between both distance to edge and matrix type groups (total number of species contributing to dissimilarities = 110 for distance to edge group and 112 for matrix group, *n* = number of species).

| Species group | Distance to edge group | | | | Matrix group | | |  |
| --- | --- | --- | --- | --- | --- | --- | --- | --- |
|  | Edge | Interior | % Contribution | *n* | Agricultural | Mining | % Contribution | *n* |
| *Foraging guilds* |  |  |  |  |  |  |  |  |
| Carnivores | 4.3 | 2.9 | 7.7 | 8 | 2.3 | 1.5 | 4.0 | 5 |
| Partial frugivores | 18.8 | 22.4 | 26.5 | 31 | 20.4 | 18.1 | 25.7 | 30 |
| Obligate frugivore | 1.6 | 1.1 | 2.2 | 3 | 1.5 | 1.2 | 2.0 | 3 |
| Granivores | 3.9 | 3.1 | 3.2 | 5 | 3.4 | 3.5 | 3.3 | 5 |
| Insectivores | 26.2 | 24.2 | 41.0 | 49 | 24.9 | 27.2 | 41.7 | 51 |
| Nectarivores | 1.5 | 1.9 | 3.5 | 4 | 4.9 | 4.3 | 5.4 | 7 |
| Omnivores | 4.2 | 4.1 | 5.5 | 6 | 4.5 | 3.9 | 5.1 | 6 |
| Raptors | 1.4 | 0.7 | 2.3 | 4 | 1.1 | 0.9 | 2.6 | 5 |
| *Habitat preference* |  |  |  |  |  |  |  |  |
| Specialists | 19.5 | 31.7 | 37.4 | 46 | 30.4 | 24.7 | 40.3 | 49 |
| Generalists | 23.8 | 23.7 | 28.7 | 36 | 30.4 | 32.0 | 43.4 | 54 |
| Visitors | 12.2 | 3.7 | 17.2 | 19 | 0.7 | 1.5 | 2.3 | 3 |
| Open country | 4.4 | 0.6 | 6.4 | 9 | 1.7 | 2.6 | 4.3 | 6 |
