# Supplementary material for: Matrix Intensification Alters Avian Functional Group Composition in Adjacent Rainforest Fragments
Source: PLoS One. 2013 Sep 13;8(9):e74852. doi: 10.1371/journal.pone.0074852 (PMC3772896; doi:10.1371/journal.pone.0074852)
Supplement: Table S2 — Significant bio-indicator species characterizing edge and interior communities. (DOCX) [file pone.0074852.s002.docx]

Table S2: Significant bio-indicator species characterizing edge and interior communities (FF=specialists, F=generalists, f=forest visitors, O=open country, AFW=widely spread throughout Africa, GCE= Guinea-Congolian endemic, UGE=Upper-Guinea endemic and RLE= threatened species; Indicator value = a quantitative index of species alliance to a classification of sites and ranges between 0 and 1)

| Species | Family | African range | Habitat  preference | Indicator value | p-value |
| --- | --- | --- | --- | --- | --- |
| ***Edge*** |  |  |  |  |  |
| *Pycnonotus barbatus* | Pycnonotidae | AFW | f | 0.68 | <0.01 |
| *Chrysococcyx klaas* | Cuculidae | AFW | F | 0.64 | <0.01 |
| *Terpsiphone rufiventer* | Monarchidae | GCE | F | 0.60 | 0.03 |
| *Camaroptera brachyura* | Sylviidae | AFW | f | 0.55 | 0.03 |
| *Halcyon senegalensis* | Alcedinidae | AFW | f | 0.54 | 0.02 |
| *Prinia subflava* | Sylviidae | AFW | f | 0.52 | 0.01 |
| *Platysteira cyanea* | Platysteiridae | GCE | f | 0.44 | 0.00 |
| *Hirundo rustica* | Hirundinidae | GCE | O | 0.39 | 0.04 |
| *Merops albicollis* | Meropidae | AFW | f | 0.38 | 0.02 |
| *Merops pusillus* | Meropidae | AFW | f | 0.38 | 0.03 |
| *Merops gularis* | Meropidae | GCE | FF | 0.31 | 0.04 |
| ***Interior*** |  |  |  |  |  |
| *Pogoniulus atroflavus* | Capitonidae | GCE | FF | 0.64 | 0.02 |
| *Phyllastrephus icterinus* | Pycnonotidae | GCE | FF | 0.59 | 0.01 |
| *Macrosphenus concolor* | Sylviidae | GCE | FF | 0.58 | 0.02 |
| *Macrosphenus kempi* | Sylviidae | UGE | FF | 0.55 | 0.01 |
| *Apalis sharpii* | Sylviidae | UGE | FF | 0.53 | 0.02 |
| *Bleda.eximia** | Pycnonotidae | RLE | FF | 0.35 | 0.04 |
| *Illadopsis.rufescens** | Timalidae | RLE | FF | 0.35 | 0.04 |
