# Supplementary material for: Matrix Intensification Alters Avian Functional Group Composition in Adjacent Rainforest Fragments
Source: PLoS One. 2013 Sep 13;8(9):e74852. doi: 10.1371/journal.pone.0074852 (PMC3772896; doi:10.1371/journal.pone.0074852)
Supplement: Table S3 — Significant bio-indicator species characteristic of remnants located in two matrix types. (DOCX) [file pone.0074852.s003.docx]

Table S3: Significant bio-indicator species characteristic of remnants located in two matrix types (FF=specialists, F=generalists, f=forest visitors, O=open country, AFW=widely spread throughout Africa, GCE= Guinea-congolian endemic, UGE=Upper-Guinea endemic and RLE= threatened species)

| Species | Family | African range | Habitat  preference | Indicator value | p-value |
| --- | --- | --- | --- | --- | --- |
| ***Agricultural*** |  |  |  |  |  |
| *Bleda syndactyla* | Pycnonotidae | GCE | FF | 0.85 | < 0.01 |
| *Streptopelia semitorquata* | Columbidae | AFW | f | 0.67 | 0.01 |
| *Phyllastrephus albigularis* | Pycnonotidae | GCE | FF | 0.63 | 0.00 |
| *Centropus leucogaster* | Cuculidae | UGE | FF | 0.62 | 0.02 |
| *Bleda canicapilla* | Pycnonotidae | GCE | F | 0.62 | 0.04 |
| *Criniger barbatus* | Pycnonotidae | GCE | FF | 0.61 | 0.01 |
| *Centropus senegalensis* | Cuculidae | AFW | f | 0.58 | 0.03 |
| *Phyllastrephus icterinus* | Pycnonotidae | GCE | FF | 0.57 | 0.02 |
| *Macrosphenus kempi* | Sylviidae | UGE | FF | 0.53 | 0.02 |
| *Polyboroides typus* | Accipitridae | AFW | f | 0.44 | 0.01 |
| *Poicephalus gulielmi* | Psittacidae | GCE | FF | 0.42 | 0.01 |
| ***Mining*** |  |  |  |  |  |
| *Pogoniulus subsulphureus* | Capitonidae | GCE | FF | 0.71 | 0.00 |
| *Merops pusillus* | Meropidae | AFW | f | 0.38 | 0.01 |
| *Cuculus clamosus* | Cuculidae | AFW | F | 0.31 | 0.04 |
| *Eurystomus gularis* | Coraciidae | GCE | F | 0.31 | 0.04 |
| *Parmoptila rubrifrons* | Estrildidae | UGE | FF | 0.31 | 0.04 |
