# Supplementary material for: Matrix Intensification Alters Avian Functional Group Composition in Adjacent Rainforest Fragments
Source: PLoS One. 2013 Sep 13;8(9):e74852. doi: 10.1371/journal.pone.0074852 (PMC3772896; doi:10.1371/journal.pone.0074852)
Supplement: Table S4 — Results from two-factor ANOVA comparing explanatory variables between distance to edge and matrix categories. (DOCX) [file pone.0074852.s004.docx]

Table S4: Results from two-factor ANOVA comparing explanatory variables between distance to edge and matrix categories

| Explanatory variable | Agricultural matrix(mean ± SD) | | Mining matrix (mean ± SD) | | *F-* statistics | | |
| --- | --- | --- | --- | --- | --- | --- | --- |
|  | Edge | Interior | Edge | Interior | Dist. edge | Matrix | Interaction |
| Large trees(counts/ha) | 1.77(1.53) | 3.72(1.65) | 0.71(0.28) | 2.12(0.82) | 15.45*** | 9.72* | 0.54 |
| Forest extent (1 km^2^) | 1.46(0.26) | 2.61(1.41) | 0.37(0.37) | 1.06(0.60) | 10.59** | 21.85*** | 0.41 |
| Fruiting trees(counts/ha) | 64(16.95) | 37(18.90) | 32.81(16.28) | 39.06(22.60) | 2.32 | 4.96* | 6.06* |

Significance codes: <0.001 '***'; < 0.01 '**' <0.05 '*'
