# Supplementary material for: Matrix Intensification Alters Avian Functional Group Composition in Adjacent Rainforest Fragments
Source: PLoS One. 2013 Sep 13;8(9):e74852. doi: 10.1371/journal.pone.0074852 (PMC3772896; doi:10.1371/journal.pone.0074852)
Supplement: Table S5 — Correlation matrix of explanatory variables. Coefficients in bold shows highly correlated variables that were excluded in the analyses. (DOCX) [file pone.0074852.s005.docx]

Table A5: Correlation matrix of explanatory variables. Coefficients in **bold** shows highly correlated variables resulting in one of the pair being excluded from the analyses.

| Explanatory variables | 1 | 2 | 3 | 4 | 5 | 6 | 7 | 8 |
| --- | --- | --- | --- | --- | --- | --- | --- | --- |
| 1. Dist. Edge | 1 |  |  |  |  |  |  |  |
| 2. Matrix | 0 | 1 |  |  |  |  |  |  |
| 3. Canopy cover | **0.7** | -0.1 | 1 |  |  |  |  |  |
| 4. Shrub density | **-0.8** | 0.3 | **-0.7** | 1 |  |  |  |  |
| 5. Fruiting trees | -0.2 | -0.2 | -0.1 | 0 | 1 |  |  |  |
| 6. Flowering trees | -0.2 | -0.2 | -0.1 | 0.1 | **0.7** | 1 |  |  |
| 7. Large trees | 0.5 | -0.2 | **0.7** | **-0.6** | 0.1 | 0.1 | 1 |  |
| 8. Forest extent (1 km^2^) | **-0.6** | 0.1 | -0.3 | 0.4 | 0.1 | 0 | -0.1 | 1 |
